# Phototoxicity of the Ethanolic Extract of *Skeletonema marinoi* for the Dermocosmetic Improvement of Acne

**DOI:** 10.3390/md22080343

**Published:** 2024-07-27

**Authors:** Jean-Baptiste Bérard, Tan-Sothea Ouk, Vincent Sol, Louise Lefoulon, Cornelia Landolt, Karine Grenier, Naima Saad, Elise Robert, Manoëlla Sibat, Nathalie Schreiber, Samuel Guenin, Laurent Picot

**Affiliations:** 1 French Research Institute for Exploitation of the Sea (IFREMER), PHYTOX, 44000 Nantes, France; louise.lefoulon@gmail.com (L.L.); elise.robert@ifremer.fr (E.R.); manoella.sibat@ifremer.fr (M.S.); nathalie.schreiber@ifremer.fr (N.S.); 2 Laboratoire des Agroressources, Biomolécules et Chimie pour l’Innovation en Santé (LABCiS), University of Limoges, UR 22722, 87000 Limoges, France; tan-sothea.ouk@unilim.fr (T.-S.O.); vincent.sol@unilim.fr (V.S.); cornelia.landolt@unilim.fr (C.L.); karine.grenier@unilim.fr (K.G.); naima.saad@unilim.fr (N.S.); 3 QIMA Life Sciences, 86160 Gençay, France; samuel.guenin@qima.com; 4 UMR CNRS 7266 LIENSs, La Rochelle Université, 17042 La Rochelle, France

**Keywords:** acne, biofilm, *Cutibacterium*, dermocosmetics, infection, microalgae, pheophorbide *a*, photodynamic therapy, pigment, *Skeletonema*, *Staphylococcus*

## Abstract

Acne is one of the most common dermatological conditions, peaking during adolescence and early adulthood, affecting about 85% of individuals aged 12–24. Although often associated with teenage years, acne can occur at any age, impacting over 25% of women and 12% of men in their forties. Treatment strategies vary depending on the severity, including the use of topical gels or creams containing benzoyl peroxide and retinoids, antibiotics, and systemic or topical isotretinoin. However, these treatments can cause irritation, allergies, and other toxic side effects. Currently, there is no natural-based alternative for antibacterial photodynamic therapy targeting acne using marine drugs or extracts. Through a bioguided screening approach, we identified the ethanol extract of *Skeletonema marinoi* as highly phototoxic against three bacterial species associated with acne—*Cutibacterium acnes*, *Staphylococcus aureus*, and *Staphylococcus epidermidis*. This extract exhibited phototoxicity in planktonic bacteria under white and red light, disrupted bacterial biofilms, reduced sebum production but also showed phototoxicity in keratinocytes, highlighting the importance of the specific targeting of treatment areas. Further investigations, including fractionation and high-resolution structural analysis, linked the observed phototoxicity to a high concentration of pheophorbide *a* in the extract. Given its notable in vitro efficacy, this extract holds promising potential for clinical evaluation to manage mild acne. This discovery paves the way for further exploration of *Skeletonema* pigment extracts, extending their potential applications beyond acne phototherapy to include dermocosmetics, veterinary medicine, and other phototherapy uses.

## 1. Introduction

Acne is a common skin condition that affects millions of individuals worldwide, characterized by the presence of inflamed sebaceous glands, clogged pores, and often the formation of pimples, blackheads, or whiteheads [1]. While typically associated with adolescence, acne can persist into adulthood and even emerge for the first time in later years, impacting both physical appearance and emotional well-being. Alongside acne, bacterial infections of the skin pose significant challenges, as they can lead to various dermatological disorders, ranging from minor irritations to severe and potentially life-threatening conditions. Among the bacteria implicated in skin infections, *Cutibacterium acnes* stands out as a major culprit in acne development [2]. This anaerobic bacterium thrives in the sebaceous glands of the skin, contributing to inflammation and the formation of acne lesions. However, aside from this species, other bacterial genera, notably *Staphylococci*, can contribute to the pathophysiology of acne as well as skin infections such as impetigo, folliculitis, and cellulitis [3,4,5].

In recent years, the rise of antibiotic resistance has emerged as a formidable obstacle in the treatment of bacterial infections. Overuse and misuse of antibiotics have fueled the development of resistant strains, rendering conventional antibiotic therapies less effective or even obsolete [6,7,8]. This alarming trend underscores the urgent need for alternative strategies to combat bacterial infections effectively. One promising avenue of research involves antimicrobial PhotoDynamic Therapy (aPDT) with photosensitizers [9,10,11,12]. This innovative approach harnesses the power of light and photosensitizers to selectively target and eliminate pathogenic bacteria through the localized production of reactive oxygen species (ROS). By administering photosensitizers topically and then exposing the infected area to light, aPDT offers a non-invasive and potentially highly effective means of treating bacterial infections. Moreover, aPDT presents several distinct advantages over traditional antibiotic treatments. In contrast to antibiotics, which may disturb the body’s natural microbiota and foster resistance, aPDT carries a lower risk of systemic side effects as it predominantly addresses localized infections. Additionally, aPDT has shown effectiveness against multidrug-resistant bacteria [13,14], effectively restricting biofilm formation [13,15] and diminishing virulence factors [16,17]. Importantly, aPDT does not induce resistance as bacteria cannot adapt to the lethal oxidative stress induced by ROS. Actually, in aPDT, the active bactericidal agent is not the photosensitizer (PS) itself but the reactive oxygen species (ROS) it generates under light illumination. These ROS exhibit high toxicity at their site of production and have a short lifespan, which prevents the development of resistance.

Despite the prevalent use of pharmaceutical-grade anticancer photosensitizers in cutaneous phototherapy [18,19], there is a noticeable absence of dermocosmetics tailored for self-managed improvement of mildly disturbed skin through a gentle aPDT approach. Consequently, there is a pressing need for cost-effective solutions to democratize the use of aPDT for the general public. While clinically developed marine drugs for acne management are lacking, certain phototoxic pigments found in marine organisms could show promise as photosensitizers for skin bacterial infection photodynamic therapy. These pigments, including porphyrins, chlorins, bacteriochlorins, and phycobiliproteins, are widely distributed in various marine microorganisms and play a crucial role in photosynthesis [20]. Notably, microalgae and cyanobacteria, known for their wide chemical diversity of pigments, offer the potential for economically viable pigment production on an industrial scale [21,22,23]. Phototoxic pigments can be present in living photosynthetic cells or produced during extraction processes [24].

In this study, during an initial assessment of marine microalgal diversity, we identified *Skeletonema marinoi* (*Skm*) as a significant producer of pigments capable of generating ROS under light exposure. *Skm* is recognized for its carefully controlled cultivation methods, adaptability for industrial use, and international approval for cosmetic purposes, making it an ideal candidate for further investigation into its phototoxic pigments [25,26,27,28] (see also Inventory of Existing Cosmetic Ingredients in China IECIC 2021 at https://www.nmpa.gov.cn/ (accessed on the 25 July 2024) and international indexation of “*Skeletonema costatum* extract” on the International Nomenclature of Cosmetic Ingredients INCI website at https://www.cirs-reach.com/Cosmetic_Inventory/International_Nomenclature_of_Cosmetic_Ingredients_INCI.html (accessed on the 25 July 2024)). The *Skm* ethanol extract exhibited significant phototoxicity against planktonic cells of *Cutibacterium acnes*, *Staphylococcus aureus*, and *Staphylococcus epidermidis*. It not only hindered biofilm formation in *S. aureus* after illumination of planktonic cells but also effectively eradicated established *S. aureus* biofilms. The degree of phototoxicity was dependent on light dosage and correlated with a high concentration of pheophorbide *a* in the extract, surpassing that found in pigment extracts from other *Skeletonema* species or *Skm* strains. Moreover, the extract demonstrated low cytotoxicity on primary keratinocytes in the absence of light and exhibited significant anti-lipogenic activity on sebocytes, two characteristics making it promising for potential application in acne treatment. The consistent production of phototoxic compounds from *Skeletonema marinoi* holds significant potential for advancing dermocosmetic formulations aimed at addressing mild to moderate acne. Additionally, investigating the broader utility of this phototoxic extract in antimicrobial therapies for humans, animals, and plants through pigment photoactivation offers promising avenues for further research and development.

## 2. Results and Discussion

### 2.1. Phototoxicity of the Skeletonema sp. Ethanol Extracts in Planktonic Bacteria

**Figure 1:**
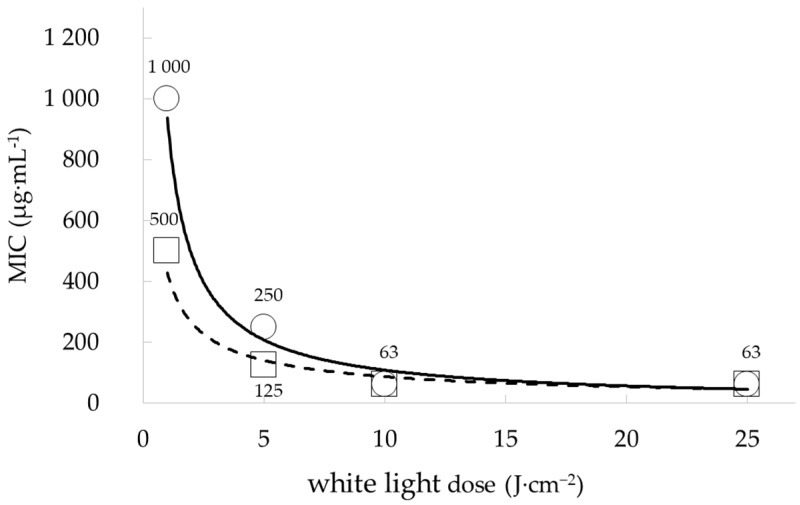
White light dose dependence of the phototoxicity of the *Skeletonema marinoi* ethanol extract in *S. aureus* planktonic cells. Illumination with white light doses of 10 J·cm^−2^ induced the maximal phototoxicity while a lower fluence (5 J·cm^−2^) increased MIC (square dots, squared regression in dashed line, *R*^2^ = 0.92) and MBC (round dots, squared regression in solid line, *R*^2^ = 0.92) values. All experiments were performed in triplicate.

The phototoxicity of the ethanolic extracts from four *Skeletonema* species (*S. marinoi, S. grethae, S. menzelii* and *S. subsalsum*) was determined on three bacterial species involved in the etiology of acne: *S. aureus*, *S. epidermidis* and *C. acnes*. The four extracts did not inhibit the growth of *C. acnes* under dark conditions (MIC > 1000 µg·mL^−1^) but were moderately phototoxic for this species under red light illumination and highly phototoxic under white light illumination (Table 1), evidencing that they contained photoactivable molecules. The *S. marinoi (Skm)* ethanol extract was the most phototoxic, giving MIC values of 125 and 6 µg·mL^−1^ in *C. acnes* under red and white light illumination, respectively (Table 1). The *Skm* ethanol extract was also highly phototoxic in *S. aureus* and *S. epidermidis*, with MIC after white light illumination of 63 and 16 µg·mL^−1^, respectively. Although the *S. grethae* ethanol extract was the most phototoxic in *S. aureus* after white light illumination (MIC = 31 µg·mL^−1^), the *Skm* ethanol extract was selected for further analysis, as it was the most active on the three bacterial species. This extract was highly phototoxic at illumination doses as low as 10 J·cm^−2^ (Figure 1) and the anaerobic species *C. acnes* was more sensitive to this phototoxicity compared to both *Staphylococcus* species (facultative aerobic-anaerobic species) after white light illumination (Table 1). The higher sensitivity of the anaerobe was consistent with the greater sensitivity of this strain to the toxic effect of ROS, compared to *Staphylococci* that have evolved efficient ROS detoxification mechanisms (antioxidant carotenoids, detoxification enzymes including superoxide dismutases, catalases and glutathione peroxidases) in response to adaptation to aerobiosis [29,30,31,32].

Based on these data, we also investigated the minimal bactericidal concentration (MBC) of the *Skm* extract on these three bacterial strains (Table 2). In *S. aureus* and *S. epidermidis*, MIC values measured at dark were lower than MBC values, suggesting that the *Skm* ethanol extract contained antibacterial molecules that could have a bacteriostatic inhibitory effect on the two strains, without killing them. In contrast, under white light illumination, the MIC values were equal or in the range of MBC values, evidencing that the growth inhibition of bacteria was related to a photobactericidal effect of the *Skm* extract.

*S. aureus* and *S. epidermidis* exhibited equivalent resistance to the photobactericidal effects of the *Skm* ethanol extract under white light exposure, both with MBC values of 63 µg·mL^−1^. However, *S. aureus* demonstrated greater resistance to growth inhibition induced by the extract at white light, with an MIC of 63 µg·mL^−1^ compared to 16 µg·mL^−1^ for *S. epidermidis*. The selection of *S. aureus* for further investigation into the activity and composition of the *Skm* ethanol extract was also influenced by its status as a model species for virulence studies, the availability of extensive data on this species, and its arsenal of virulence factors, making it a more problematic skin pathogen than *S. epidermidis*.

### 2.2. Phototoxicity of the Skeletonema marinoi Ethanol Extract in Planktonic S. aureus Depends on Illumination Energy

Confirming the light-dependence toxicity of the *Skm* extract, a correlation of phototoxicity with the white light dose was observed in *S. aureus* planktonic cells, with MIC and MBC increasing at low photoactivation energy (Figure 1). A light energy level of 10 J·cm^−2^ proved adequate to elicit the maximum phototoxic effect on this species (Figure 1).

### 2.3. Phototoactivation of the Skeletonema marinoi Ethanol Extract Inhibits the Formation of S. aureus Biofilms and Destroys Established Biofilms

We explored the potential of the *Skm* ethanol extract to influence the production of biofilms by planktonic *S. aureus* or to eradicate established biofilms through photoactivation.

In the initial experiment, planktonic bacteria were treated with varying concentrations of the *Skm* ethanol extract, either in darkness or under white light illumination (total fluence of 25 J·cm^−2^), before biofilm formation. This treatment was compared to a positive control using bleach. The biofilm formed 24 h post-treatment was visualized using crystal violet (CV) staining. In a subsequent experiment, planktonic bacteria were allowed to form a dense biofilm for 48 h before undergoing the same treatment.

**Figure 2:**
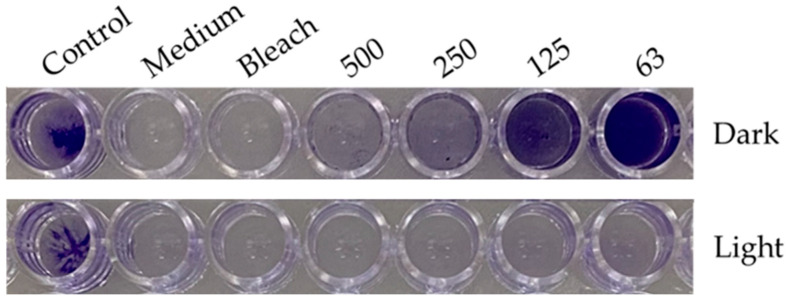
Photoactivation of the ethanol extract of *Skeletonema marinoi* induces a dose-dependent inhibition of *Staphylococcus aureus* biofilm formation. *S. aureus* planktonic cells were treated with control medium (Control), bleach, or increasing concentrations of *Skm* ethanolic extract under both dark conditions (upper line) or white light illumination (total fluence of 25 J·cm^−2^, lower line). The “Medium” column indicates a negative control with no bacteria. Following a 24-h incubation at 37 °C, the residual biofilm was stained with crystal violet (CV) and quantified through UV-Vis spectrophotometry at 595 nm after CV solubilization with acetic acid. All experiments were performed in triplicate.

**Figure 3:**
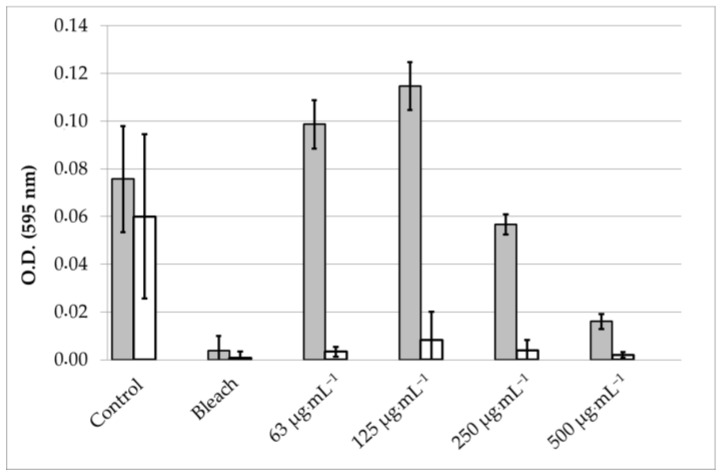
Photoactivation of the ethanol extract of *Skeletonema marinoi* induces a dose-dependent inhibition of *Staphylococcus aureus* biofilm formation. *S. aureus* planktonic cells were treated with control medium, bleach, or increasing concentrations of *Skm* ethanolic extract under both dark conditions (grey bars) or white light illumination (total fluence of 25 J·cm^−2^, white bars). Following a 24-h incubation at 37 °C, the residual biofilm was stained with crystal violet (CV) and quantified through UV-Vis spectrophotometry at 595 nm after CV solubilization with acetic acid. All experiments were performed in triplicate.

**Figure 4:**
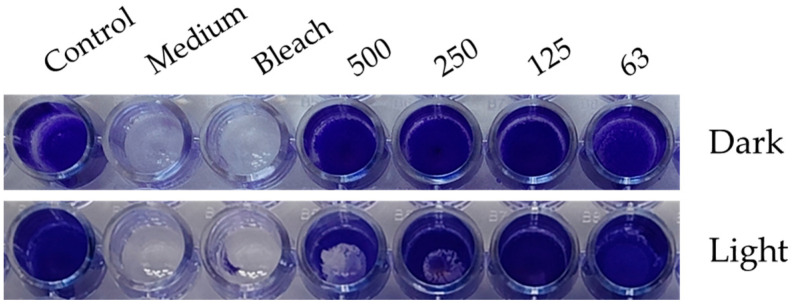
Eradication of *S. aureus* mature biofilm by the *Skm* ethanol extract after photo-illumination with white light. Photo-illumination with white light doses of 25 J·cm^−2^ induced a partial eradication of *S. aureus* mature biofilm at concentrations of 250 and 500 µg·mL^−1^. All experiments were performed in triplicate independent assays.

**Figure 5:**
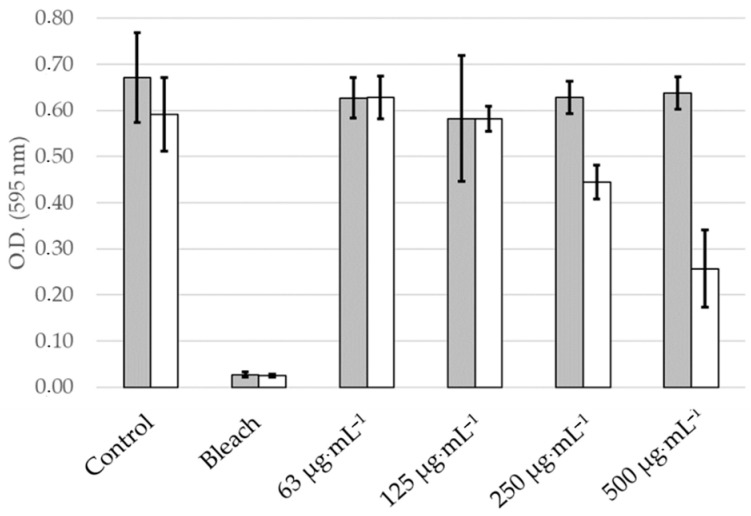
Phototoxicity of the *Skm* ethanol extract in established bacterial biofilms. *S. aureus* cells were allowed to form biofilms covering the entire well surface for 48 h in plastic microplates before treatment with bleach or *Skm* ethanol extract at doses ranging from 63 to 500 µg·mL^−1^ at dark (grey bars) or after light illumination (total fluence of 25 J·cm^−2^, white bars). The biofilm remaining after treatment was colored with Coomassie blue and quantified by spectrophotometry at 595 nm. All experiments were performed in triplicate independent assays.

Under dark conditions, inhibition of biofilm formation was observed at *Skm* ethanol extract doses exceeding 125 µg·mL^−1^. This inhibition may be attributed to the presence of surfactant molecules in the extract, disrupting the polymeric matrix favoring bacterial cell attachment to the plastic surface, or to the presence of molecules directly toxic in planktonic bacterial cells. These compounds possibly include membrane lipids (such as tri-, di- and monoacylglycerols, fatty acids including omega-3 and omega-6 fatty acids), glycolipids, carotenoids, chlorophylls and their derivatives (including hydroxy-derivatives formed during ethanolic extraction [21]), as well as sterols [33]. In contrast, white light illumination of the *Skm* ethanol extract resulted in almost complete inhibition of *S. aureus* biofilm formation, even at concentrations as low as 63 µg·mL^−1^ (Figure 2 and Figure 3). Additionally, in a particularly interesting manner due to the resistance of biofilms to conventional treatments, the *Skm* ethanol extract partially eradicated the 48-h mature biofilm of *S. aureus* at concentrations of 250 and 500 µg·mL^−1^ after illumination with white light at 25 J·cm^−2^, while no eradication was observed in the dark (Figure 4 and Figure 5).

### 2.4. Toxicity and Phototoxicity of the Skeletonema marinoi Ethanol Extract in Primary Normal Human Epidermal Keratinocytes (NHEK)

As the phototoxic extract is intended for application to the skin for dermocosmetic purposes, its toxicity at dark and phototoxicity were evaluated in human keratinocytes. A probable phototoxicity in human keratinocytes was anticipated, given that oxygen singlet and reactive oxygen species (ROS) exert non-specific oxidative actions in both prokaryotic and eukaryotic cells. These actions encompass amino acid oxidation, protein fragmentation and cross-linkage, lipoperoxidation of membrane lipids, as well as the oxidation of nitrogenous bases in nucleic acids [34,35].

**Figure 6:**
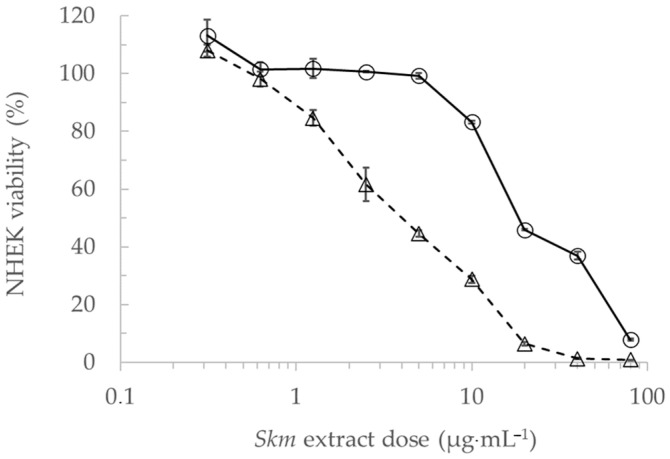
Cytotoxicity in the dark (solid line, round dots) and phototoxicity (dashed line, triangular dots) after 25 J·cm^−2^ white light illumination of the *Skeletonema marinoi* ethanol extract in primary cultures of Normal Human Epidermal Keratinocytes (NHEK). Cells were grown in cell culture medium and treated at obscurity or in the presence of white light with the *Skm* ethanol extract at doses ranging from 0.3 to 80 µg·mL^−1^ in cell culture medium. Cell viability was then measured using the MTT assay. All experiments were performed in triplicate.

Primary NHEK cells were grown in microplates and exposed to increasing concentrations of the extract in the range of the phototoxic doses (0–500 µg·mL^−1^), in the dark or under white light at a fluence of 25 J·cm^−2^. Cell viability was determined using the MTT colorimetric cell viability assay. The extract exhibited moderate cytotoxicity in the absence of light, displaying an IC_50_ of 19 µg·mL^−1^ in cell culture medium. However, when exposed to white light at 25 J·cm^−2^, it demonstrated high phototoxicity, with an IC_50_ of 4 µg·mL^−1^ (Figure 6). When compared to *Staphylococcus aureus*, primary NHEK cells exhibited heightened sensitivity to both dark toxicity and phototoxicity of the extract. This underscores the potential risk of adverse effects in the skin surrounding acne lesions targeted for treatment. Nevertheless, it appears straightforward to effectively reduce this risk by carefully determining the extract concentration in a dermocosmetic formulation and accurately applying the cream or gel to the specific acne-affected area.

### 2.5. Antilipogenic Activity of the Skeletonema marinoi Ethanol Extract in the Human Sebocyte Cell Line SEBO662AR

Lipogenesis is a pivotal factor in the pathogenesis of acne, as the excessive production of sebum by sebaceous glands can prompt the obstruction of hair follicles [36]. This, in turn, fosters the growth of the anaerobic bacterium *Cutibacterium acnes*, leading to localized inflammation and the development of inflammatory acne lesions like papules, pustules, and nodules. Hormones, particularly androgens such as testosterone, play a crucial role in stimulating lipogenesis, elucidating the connection between sebum production and acne, which is commonly observed during adolescence and puberty. Consequently, it seemed pertinent to assess the antilipogenic activity of the *Skeletonema marinoi* ethanol extract in human sebocytes, at sub-toxic concentrations.

**Figure 7:**
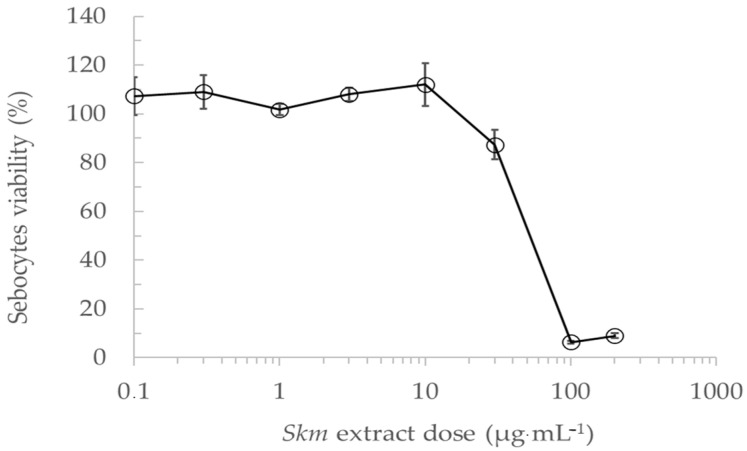
Determination of sub-toxic concentrations of the *Skeletonema marinoi* ethanol extract at dark in sebocytes. The SEBO662AR human sebocyte cell line was cultured and exposed for 7 days to increasing concentrations of *Skm* extract in the absence of light. This initial step aimed to identify sub-toxic concentrations for subsequent lipogenesis inhibition assessment. Results indicate the absence of cytotoxicity on sebocytes within the 0.1–10 µg·mL^−1^ concentration range. Cell viability was measured using the WST-8 assay. All experiments were performed in triplicate.

The SEBO662AR human sebocyte cell line was cultured and exposed for 7 days to increasing concentrations of the *Skeletonema marinoi* ethanol extract at dark, to determine in a first step the sub-toxic concentrations to be used for the determination of lipogenesis inhibition. The *Skm* ethanol extract showed no cytotoxicity on sebocytes in the 0.1–10 µg·mL^−1^ concentration range (Figure 7).

**Figure 8:**
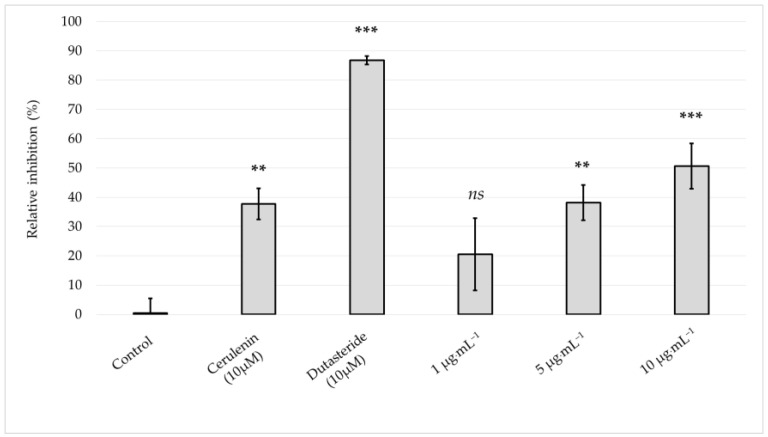
Sub-toxic concentrations of the *Skeletonema marinoi* ethanol extract inhibit lipogenesis in SEBO662AR human sebocytes. Cultured cells were exposed to increasing concentrations of *Skeletonema marinoi* ethanol extract, within a low toxicity range (1–10 µg·mL^−1^) and lipogenesis was assessed using a BODIPY^®^ 493/503 probe. The lipogenesis inhibitors cerulenin 10 µM and dutasteride 10 µM respectively induced a highly and extremely significant inhibition of lipogenesis, as compared to control conditions. The *Skm* ethanol extract induced a highly significant lipogenesis inhibition at 5 µg·mL^−1^ and extremely significant inhibition at 10 µg·mL^−1^. *p* value: ns ≥ 0.05, not significant; ** = 0.001 to 0.01, highly significant; *** ≤ 0.001, extremely significant.

**Figure 9:**
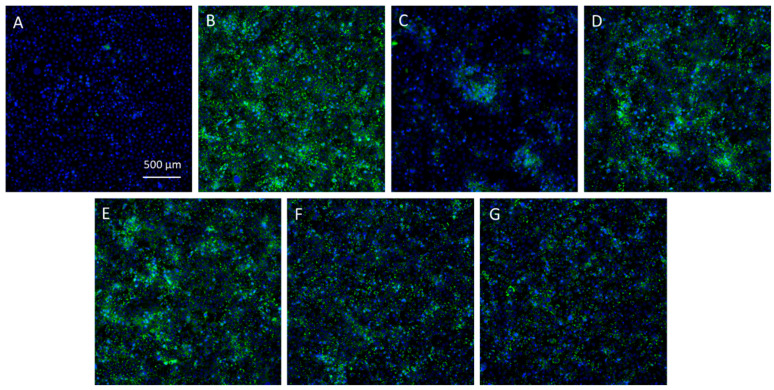
Microphotographs of SEBO662AR human sebocytes. Intracellular lipid droplets were labelled with the fluorescent probe BODIPY^®^ 493/503 before fluorescence imaging (magnification ×20). The cells were either unstimulated (**A**) or stimulated with a lipogenic mix containing Dihydrotestosterone, Vitamin D_3_, Vitamin C, insulin, and Ca^2+^ ions (confidential concentrations, QIMA Life Sciences lipogenesis inhibition assay) (**B**–**G**), followed by treatment with control medium (**B**), dutasteride at 10 µM (**C**), cerulenin at 10 µM (**D**), or the *Skm* ethanol extract at concentrations of 1 (**E**), 5 (**F**), or 10 (**G**) µg·mL^−1^. Cell nuclei were stained with Hoechst (blue labeling), while green labeling indicates lipogenesis detected using the BODIPY^®^ probe.

The effect of the *Skm* ethanol extract on inhibiting lipogenesis under light-free conditions was assessed by determining the relative percentage of inhibition of lipid droplet formation in sebocytes. The *Skm* ethanol extract induced highly significant and extremely significant lipogenesis inhibition at 5 and 10 µg·mL^−1^, respectively (Figure 8 and Figure 9).

### 2.6. Analysis of the Pigment Composition of the Skeletonema marinoi Ethanol Extract

**Figure 10:**
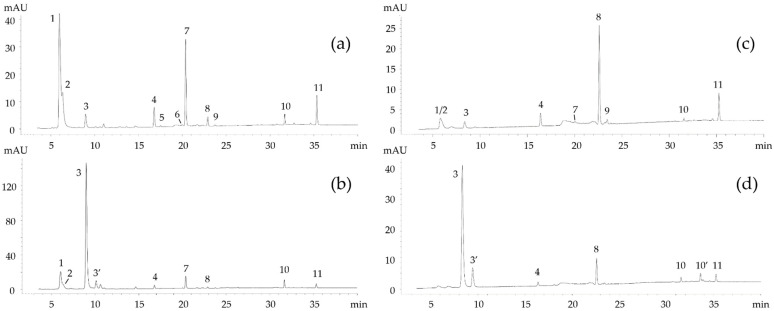
Reverse Phase HPLC chromatogram of *Skeletonema marinoi* pigments and derivatives in in vivo planktonic cells at 450 nm (**a**) (maximum absorbance wavelength of chlorophylls and carotenoids) and 405 nm (**b**) (maximum absorbance wavelength of pheophorbide *a*), and in ethanol extract at 450 nm (**c**) and 405 nm (**d**). The twelve major detected peaks, from the most polar to the most apolar, corresponded to (1) chlorophyll *c2*, (2) chlorophyll *c1*, (3) pheophorbide *a*, (3′) pheophorbide *a* isomer, (4) *trans*-fucoxanthin, (5) Neoxanthin, (6) *cis*-fucoxanthin, (7) diadinoxanthin, (8) diatoxanthin, (9) zeaxanthin, (10) chlorophyll *a,* (10’), pheophytin *a*, and (11) β,β-carotene. High peaks of pheophorbide *a* can be observed in the chromatograms of ethanol extracts.

To establish a connection between the *Skm* ethanol extract’s phototoxic activity and its composition, and to pinpoint the molecules responsible for its phototoxic effects, an analysis of the pigment composition of both *Skm* planktonic living cells and the *Skm* ethanol extract was conducted. To determine the pigment composition from *Skm* planktonic living cells, the SCOR/UNESCO reference method outlined by Van Heukelem and Thomas [37] was used, with slight modifications detailed in Serive and colleagues [23] aimed at improving accuracy and precision. This approach relies on pigment extraction using acetone and is considered the most precise method for analyzing the pigment composition of living microalgae without causing degradation. Additionally, RP-HPLC and UPLC-MS analyses of pigment extracts in ethanol, equivalent to the *Skm* ethanol extract composition, were utilized to confirm the pigment composition of the active extract [21,23]. It is well known that using ethanol as the extraction solvent introduces the possibility of chemical or enzymatic transformations, potentially leading to pigment degradation, especially chlorophyll *a* dephytylation and demetallation [21,24]. Therefore, comparing the two methods allows conclusions to be drawn regarding potential pigment degradation and chemical or enzymatic transformations during ethanolic extraction. As depicted in Figure 10, chromatograms of pigments extracted from *Skm* planktonic living cells and ethanol extract revealed 12 prominent peaks absorbing at 450 nm (indicative of chlorophylls and carotenoids) and 405 nm (the maximal absorption wavelength for pheophorbide *a*). These 12 pigments and derivatives were definitively identified as follows: (1) chlorophyll *c2*, (2) chlorophyll *c1*, (3) pheophorbide *a*, (3′) pheophorbide *a* isomer, (4) *trans*-fucoxanthin, (5) Neoxanthin, (6) *cis*-fucoxanthin, (7) diadinoxanthin, (8) diatoxanthin, (9) zeaxanthin, (10) chlorophyll *a*, and (11) β, β-carotene. Identification was based on polarity, taxonomic consistency in *Skeletonema marinoi* pigment composition, and high-resolution mass spectrometric analysis (Figure 10 and Table 3). Additional data regarding absorption spectra, maximal absorption wavelengths, and band III/II ratios for carotenoids were considered when available. Additionally, where feasible, comparison with pigment standards was employed for confirmation.

Although all identified pigments in the *Skm* ethanol extract may contribute to its phototoxicity, with the exception of fucoxanthin, which has been described as photoprotective [38,39,40], it is noteworthy that the extract was particularly enriched in pheophorbide *a*, pheophorbide *a* isomer, and the derivative 13-OH pheophorbide *a*. Pheophorbide *a* was detected as giving the most abundant ions during the MS analysis. This pheopigment, derived from chlorophyll breakdown, has been previously reported to exhibit significant phototoxicity upon exposure to light in the presence of oxygen [41,42,43,44]. When pheophorbide *a* absorbs photons, it enters an electronically excited state. In the presence of molecular oxygen, this excited state can undergo various photochemical reactions, resulting in the generation of reactive oxygen species (ROS) including singlet oxygen (^1^O_2_) and free radicals such as superoxide anion (O_2_^•−^) and hydroxyl radicals (•OH). The phototoxic effects of pheophorbide *a* have been demonstrated across a range of biological systems, including cancer cell cultures and microorganisms [45]. Consequently, its potential use has been considered for antitumoral and antibacterial phototherapy applications. We determined that the MIC of pure pheophorbide *a* for the photoinduced growth inhibition of the *S. aureus* strain used in this study was 1.95 µg·mL^−1^, compared to 62.5 µg·mL^−1^ for the raw *Skm* ethanol extract. This indicated that the extract was only 32 times less active than pure pheophorbide *a*.

From a taxonomic perspective, the high concentration of pheophorbide *a* and its derivatives in the *Skm* pigment composition suggests more than just chlorophyll *a* degradation during pigment extraction; it indicates their likely occurrence in living cells. This conclusion is supported by the demonstration that this pigment derivative is detected in high amounts in the planktonic living cells using the reference SCOR/UNESCO method developed by Van Heukelem and Thomas. The higher concentration of pheophorbide *a* in *Skm* extracts from living planktonic cells may be linked to elevated expression levels of chlorophyllases in this species [46,47].

To test the hypothesis suggesting a correlation between the phototoxicity of the extract and elevated levels of pheophorbide *a* in the species, various methodologies were employed. Initially, a bioguided fractionation of the *Skm* extract was conducted to demonstrate that the most phototoxic sub-fractions contained pheophorbide *a* or its derivatives. Additionally, the pigment composition of *Skm* was compared to that of closely related *Skeletonema* species to determine if *Skm* contained a higher amount of pheophorbide *a*. Then, a principal component analysis (PCA) was performed to investigate the correlation between the phototoxicity of the pigment extract and the concentration of pheophorbide *a*.

### 2.7. Bioguided Fractionation of the Skeletonema marinoi Ethanol Extract

**Figure 11:**
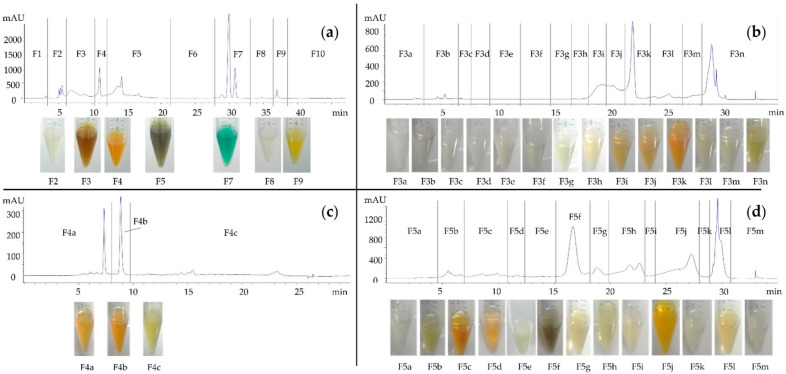
Preparative Reverse Phase HPLC Fractionation of the *Skm* ethanol extract into 10 fractions (F1 to F10 (**a**)) and sub-fractions (F3a to F3n (**b**), F4a to F4c (**c**), F5a to F5m (**d**)). The fractionation process utilized a carefully designed eluants gradient to optimize resolution for the purification of peaks (410 nm). All fractions were tested for phototoxicity under white light against *S. aureus* to continue the bioguided fractionation. Out of the 10 primary fractions collected, F3, F4, and F5 demonstrated phototoxic effects on *S. aureus* and were thus selected for a subsequent sub-fractionation.

The Skm ethanol extract underwent fractionation into 10 fractions (F1 to F10) using preparative HPLC (Figure 11), followed by testing for phototoxicity under white light against *S. aureus*. The fractionation process involved a meticulously designed eluants gradient to optimize resolution for the separation of pigments. Fractions F1, F6, and F10 were excluded from further analysis as they did not contain any visible peaks on the chromatogram.

Each sub-fraction underwent testing for bacterial growth inhibition under white and red illuminations and at dark against *S. aureus* cultivated on agar plates (Table 4). A sub-fraction was considered active (+) if it induced inhibition of bacterial growth around the deposition zone following treatment, and phototoxic if it was active under photoactivation but inactive at dark.

The exclusive phototoxicity observed in subfractions F5f and F5g was attributed to the presence of a combination of pheophorbide *a*, pheophorbide *a* isomer, and 13 OH-pheophorbide *a*. This definitive identification underscores the role of these compounds as the primary contributors to the extract’s phototoxic effects. Moreover, subfraction F3j contained a mixture of all-*trans* and *cis*-fucoxanthin, while F4b contained alloxanthin. These carotenoids have been previously documented in the literature for their antibacterial activity [48], although their potential phototoxicity cannot be assumed. Further investigation is warranted to ascertain their exact phototoxicity and biological or photophysical mechanisms involved in their observed antibacterial activity.

### 2.8. Correlation of Phototoxicity and Pheophorbide a Content through Principal Component Analysis in Various Species of Skeletonema and Phenotyping of the Pigment Contents among Different Strains of Skeletonema marinoi

As presented previously, the phototoxicity of ethanol extracts obtained from various *Skeletonema* species exhibited considerable variability (Table 1). Pigment dosages and principal component analysis (PCA) were conducted to validate that these differences in phototoxicity were associated with differences in pheophorbide *a* content. To explore further whether this phototoxicity was specific to the *Skm* strain investigated in this study, multiple strains of the *Skeletonema marinoi* species were phenotyped and compared for their pigment contents.

As illustrated in Table 5, the massic concentration of pheophorbide *a* in *Skm* strain IFR-855 cells was found to be 11.8 times greater than the concentration in *S. grethae*, and no detectable presence was observed in *S. subsalsum* and *S. menzelii*. This led us to conclude that the *Skeletonema marinoi* strain IFR-855 employed in our study displayed a notably high capacity for producing pheophorbide *a*.

**Figure 12:**
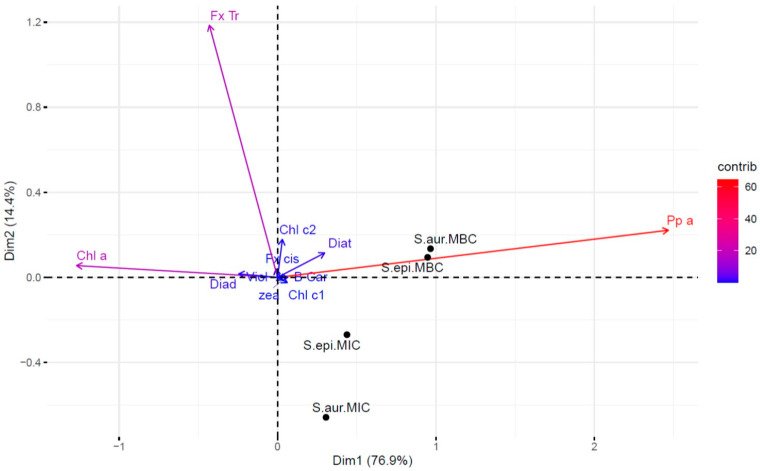
Principal Component Analysis (PCA) highlighting the variation in pigment content among extracts of the four *Skeletonema* species (*Skeletonema marinoi*, *Skeletonema grethae*, *Skeletonema subsalsum*, and *Skeletonema menzelii*) in relation to the Minimum Inhibitory Concentrations (MICs) and Minimum Bactericidal Concentrations (MBCs) measurements against *S. aureus* and *S. epidermidis*. Pigment contributions to the variation between the extracts are depicted by arrows, color-coded from weak (blue) to strong (red). Pigments included are: Chl_c2 (chlorophyll *c*2), Chl_c1 (chlorophyll *c*1), Chl_a (chlorophyll *a*), Pp_a (pheophorbide *a*), Fx_cis (*cis*-fucoxanthin), Fx_Tr (all-*trans* fucoxanthin), Diad (diadinoxanthin), Diat (diatoxanthin), Viol (violaxanthin), zea (zeaxanthin), and B_Car (β, β-carotene). Black dots represent MIC and MBC variations under white light illumination conditions (25 J·cm^−2^), with labels indicating the MIC on *S. epidermidis* (S.epi.MIC), MBC on *S. epidermidis* (S.epi.MBC), MIC on *S. aureus* (S.aur.MIC) and MBC on *S. aureus* (S.aur.MBC).

A Principal Component Analysis (PCA) was employed to correlate the pigment compositions of the total extracts obtained from *Skeletonema marinoi*, *S. grethae*, *S. subsalsum*, and *S. menzelii* (as detailed in Table 5), to the respective Minimum Inhibitory Concentrations (MICs) and Minimum Bactericidal Concentrations (MBCs) found against *S. aureus* and *S. epidermidis* under white light conditions (as detailed in Table 1) (Figure 12).

**Figure 13:**
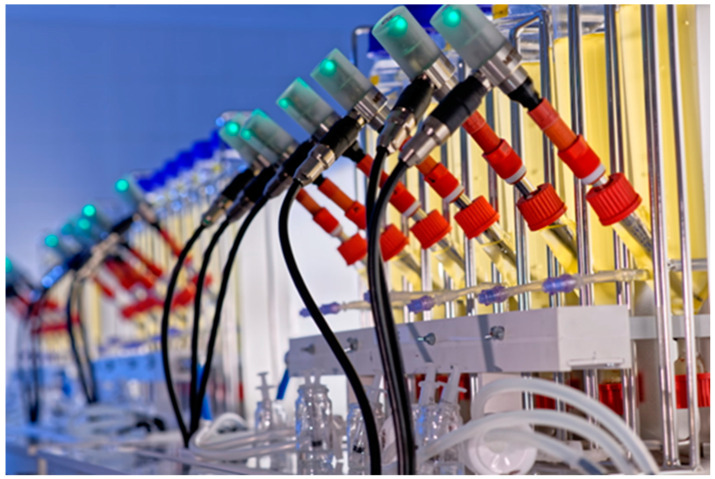
Culture of four strains of *Skeletonema marinoi* using a phenotyping bench specially designed to grow microalgae under highly homogeneous conditions of temperature, pH and light. Four non-axenic *Skm* strains were grown in 400 mL photobioreactors in batch mode in triplicate independent assays. Culture conditions consisted of 35‰ salinity sterile seawater enriched by Walne (“Conway”) medium and silica, temperature and pH were regulated at 20 °C and 8.00, respectively, and a continuous white light irradiance was set at 200 µmol.m^−2^.s^−1^. Copyright: IFREMER. O. Dugornay.

The first and second component of the PCA explained 91% of the variance of the pigment content among the *Skeletonema marinoi*, *Skeletonema grethae*, *Skeletonema subsalsum*, and *Skeletonema menzelii* extracts. Both components highlighted pheophorbide *a*, chlorophyll *a* and all-*trans* fucoxanthin as the pigments contributing to the greatest concentration variations among the extracts (Table 5), while chlorophyll *c*1, chlorophyll *c*2, *cis*-fucoxanthin, diadinoxanthin, diatoxanthin, violaxanthin, zeaxanthin, and β-carotene, with the lowest values, did not significantly vary between the extracts, all being clustered in the center of the PCA graph with poor contribution to variability. PCA results set the most significant correlation of pheophorbide *a* content with antibacterial activity against *S. aureus* and *S. epidermidis*, particularly emphasizing the role of pheophorbide *a* in Minimum Bactericidal Concentrations (MBCs). Accordingly, pheophorbide *a* was also validated as the principal molecule explaining phototoxicity through the bioguided fractionation. Conversely, chlorophyll *a* exhibited anti-correlation with antibacterial activity. Fucoxanthin demonstrated a lack of correlation to antibacterial activity in the first component of the PCA, and an anti-correlation with the MICs on *S. aureus* and *S. epidermidis* considering the second component. However, fucoxanthin was identified through the bioguided fractionation as one of the pigments possessing antibacterial properties. Due to minimal variation in fucoxanthin content among the extracts of *S. marinoi*, *S. grethae*, and *S. subsalsum*, PCA failed to adequately describe a correlation of its concentration with antibacterial activity. Moreover, the anticorrelation of fucoxanthin is strongly weighted by the low antibacterial activity found for *S. menzelii*, even though this species contains the highest abundance of this pigment. Given the substantial contribution of pheophorbide *a* to the PCA results, its photosensitizing activity confirmed by the bioguided fractionation, and its high abundance in the *S. marinoi* extract exhibiting the lowest MIC and MBC values, we investigated whether a high pheophorbide *a* cell content was a shared characteristic among different strains of the *marinoi* species or unique to the IFR-855 strain. For this purpose, a phenotyping bench system comprising 12 photobioreactors, each meticulously controlled for irradiance, temperature, and pH, was employed to ensure uniform growth conditions across all strain cultures, conducted in triplicate (Figure 13). Four strains were selected among referenced isolates: IFR-855 (strain used in this study to produce the phototoxic extract), AC-714, CCAP 1077/5, and SAG 19.99. According to the traceability of the origin of the isolations, the four strains come from two distinct isolates maintained in four different locations during decades. CCAP 1077/5 and SAG 19.99 strains were isolated from Long Island Sound (Milford Harbor, CT, USA) in 1956, while strains IFR-855 and AC-714 were isolated in the Bouin polder (Baie de Bourneuf, Vendée, France) in the early 1980s. CCAP 1077/5 and SAG 19.99 are maintained in the Culture Collection of Algae and Protozoa (UK) and in the Culture Collection of Algae at Göttingen University (DE), respectively. IFR-855 and AC-714 strains are maintained in the IFREMER Nantes facility (FR) and in the Algobank, Université de Caen Normandie (FR), respectively. Taxonomic molecular analysis confirmed that the four strains belonged to the species *Skeletonema marinoi*, both with respect to 18S ribosomal sequences (100% to 99.9% homology) and ITS ribosomal sequences (100% to 99.8%) (Supplementary Data S1).

**Figure 14:**
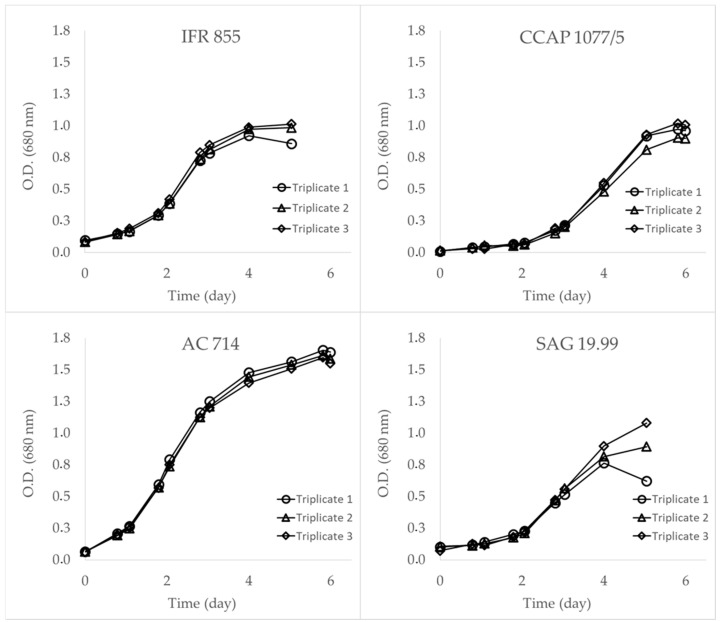
Growth curves illustrating the growth dynamics of the four distinct strains of *Skeletonema marinoi* over time. Biomass was monitored using optical density (O.D.) at 680 nm. The variations in growth patterns among the strains are depicted, showcasing differences in growth rates, and maximal O.D. at the stationary phase. Biomass was sampled at the beginning of the stationary phase for pigment analysis (Van Heukelem method) and dry mass determination after freeze-drying.

Each of the four strains exhibited typical exponential growth and ultimately reached stationary phases within a span of 4 to 5 days (Figure 14). IFR-855, AC-714, and CCAP 1077/5 cultures demonstrated a high reproducibility within triplicates, while SAG 19.99 triplicates slightly diverged during the last sampling day at the stationary phase. This robust set of cultures enabled a precise phenotyping of the pigments.

The analysis of pigment contents revealed that all four *S. marinoi* strains exhibited notably high levels of pheophorbide *a*, underscoring the species’ inclination towards producing this particular pigment. Notably, the IFR-855 and AC-714 strains demonstrated higher concentrations of pheophorbide *a* compared to CCAP 1077/5 and SAG 19.99 strains (Table 6). Furthermore, in a very noticeable way, the ratio of pheophorbide *a* to chlorophyll *a* was markedly elevated in the SK-855 strain compared to the other three strains. This finding reaffirmed the distinct propensity of the SK-855 strain to convert chlorophyll *a* into pheophorbide *a*, suggesting a potential correlation between the high phototoxicity of its pigment extract and the combination of elevated pheophorbide *a* concentration with diminished chlorophyll *a* concentration. Interestingly, the SK-855 strain is also characterized by a low content in fucoxanthin, chlorophyll *c*1 and chlorophyll *c*2, involved in the fucoxanthin chlorophyll a/c light-harvesting complex in diatoms. Diatoxanthin and diadinoxanthin, which contribute to the protective non-photochemical quenching of chlorophyll *a* through their epoxidation/de-epoxidation cycle, were found in comparable quantities in the four strains, with the exception of CCAP 1077/5, which contained lower quantities, particularly in diatoxanthin. Based on this analysis of pigment content and cell function, strain IFR-855 confirmed a distinct phenotype when grown under conditions strictly comparable to other strains, although it is likely to vary under other light conditions.

## 3. Conclusive Remarks

Bacterial infections and antibiotic resistance pose significant global health challenges, impacting both human and animal well-being. The misuse and overuse of antibiotics have led to the emergence of multidrug-resistant bacteria, rendering many conventional treatments ineffective and necessitating the use of antibacterial treatments with undesirable side effects, particularly for managing mild to moderate skin infections and dysbiosis.

The investigation into the phototoxicity of the ethanolic extract of *Skeletonema marinoi* revealed its promising potential for improving acne care through targeted antibacterial photodynamic therapy (aPDT). Our data demonstrated its significant phototoxic effects against three key bacterial species associated with acne—*Cutibacterium acnes*, *Staphylococcus aureus*, and *Staphylococcus epidermidis*—under both white and red light exposure. *C. acnes*, as an anaerobic bacterium, exhibited greater sensitivity to the phototoxic effects compared to the facultative anaerobes *S. aureus* and *S. epidermidis*, in accordance with the differing abilities of these bacteria to detoxify reactive oxygen species (ROS) generated when the extract’s pigments are activated by light. The extract also showed potential in mitigating sebum production, positioning it as a suitable candidate for further clinical evaluation as a natural alternative for acne treatment. Minimal inhibitory concentration (MIC) and minimal bactericidal concentration (MBC) assessments highlighted the extract’s potent antibacterial properties, particularly under white light illumination. The correlation between phototoxicity and light dosage suggested that effective antibacterial activity with limited adverse side effects for skin could be optimized by adequately adjusting light intensity. Additionally, the study emphasized the importance of applying the phototoxic extract specifically to the targeted treatment area and adjusting the extract concentration in a suitable galenic formulation to limit adverse phototoxicity in skin cells, in view of future clinical evaluation.

Biofilms represent a significant challenge in treating bacterial infections due to their resistance to conventional treatments. Under dark conditions, the *S. marinoi* ethanol extract induced partial inhibition of biofilm formation, likely due to the presence of direct antibacterial molecules or surfactant molecules that may disrupt the polymeric matrix favoring bacterial attachment. White light photoactivation significantly enhanced the extract’s efficacy, nearly completely inhibiting biofilm formation by planktonic cells at low concentrations and allowing the partial eradication of established biofilms. The ability to disrupt and eradicate biofilms is particularly important for acne management, as biofilms contribute to the persistence and recurrence of acne lesions, associated with induction of resistance to treatments.

Bioguided fractionation and high-resolution structural analysis identified pheophorbide *a* and its derivatives as the primary contributors to the extract’s phototoxicity, providing a foundation for optimizing the extract’s formulation for clinical use. Efforts should be focused on maximizing its antibacterial efficacy while minimizing potential side effects. Future research should prioritize clinical evaluations to establish the extract’s safety and efficacy in vivo, helping to determine the optimal dosage, formulation, application, and photoactivation methods for treating mild to moderate acne. Additionally, exploring the industrial production of the extract and understanding the biosynthesis of phototoxic compounds during microalgal cell growth will be crucial steps for scaling up its use. This includes optimizing cultivation conditions for *Skeletonema marinoi* to enhance the yield of phototoxic pigments, ensuring consistent production quality at an industrial scale, and optimizing extraction methods and formulation to ensure a consistent and high-quality output of a final standardized product for dermocosmetic use.

Finally, the discovery and development of marine pigment-based phototoxic extracts for dermocosmetics, veterinary medicine, and phytopharmaceutical interventions could open new markets and provide sustainable, natural, effective, and eco-friendly alternatives to conventional treatments, revolutionizing current treatment paradigms.

## 4. Materials and Methods

### 4.1. Microalgae Collection and Cultures

The *Skeletonema* genus was selected for metabolites extraction as these diatoms combine several advantages to make them good sources of photosensitizers for dermocosmetic applications. The genus contains several closely related species than can be screened for their production of photosensitizers, giving a rationale between activity and pigment composition and abundance. The genus was also described for its high expression of chlorophyllases, the enzymes that degrade chlorophyll *a* to chlorophyllide *a*, the precursor for pheophorbide *a* and its metabolites, that are highly phototoxic. In this sense, data reported in a previous independent research project focused on tumor phototherapy (ANR Photomer Emergence 2008—https://anr.fr/Projet-ANR-08-EBIO-0016, accessed on the 25 July 2024) highlighted the extract’s capacity to generate reactive oxygen species (ROS) under photoactivation by red LASER light (660–680 nm). Additionally, species from the *Skeletonema* genus are abundant in the oceans, available in biobanks, easy to grow in cheap standardized conditions in photobioreactors and already authorized for the production of extracts that can be sold internationally, opening the way to potential important and fast market opportunities for dermocosmetic applications [49]. *Skeletonema marinoi* (IFR-855), *S. subsalsum* (SAG 8.94), *S. grethae* (CCAP 1077/3) and *S. menzelii* (CCMP 787) strains were provided by IFR-IFREMER institute, SAG-University of Göttingen, CCAP-Scottish Association for Marine Science and CCMP-National Center for Marine Algae and Microbiota Bigelow Laboratory for Ocean Sciences. *Skeletonema marinoi* CCAP 1077/5 and SAG 19.99 strains were obtained from the Culture Collection of Algae and Protozoa (UK) and in the Culture Collection of Algae at Göttingen University (DE), respectively. *Skeletonema marinoi* IFR-855 and AC-714 were obtained from the IFREMER Nantes facility (FR) and from the Algobank, Université de Caen Normandie (FR), respectively. Microalgae were grown in batch mode in sterile seawater enriched with Walne’s medium and silica, except for *Skeletonema subsalsum* which was grown in sterile freshwater. Batch cultures were maintained at 20 °C under continuous light using fluorescent lamps (Philips TLD 58W 865). Cultures were mixed by bubbling 0.22 μm filtered air. Photobioreactor volumes varied from 400 mL to 50 L depending on the experimental and production needs. For 400 mL to 10 L cultures, cells were harvested by centrifugation with a laboratory rotor centrifuge (5000× *g* for 20 min at 4 °C) while larger volumes were harvested with a disc-stack centrifuge (GEA WESTFALIA SEPARATOR OTC 2-02-137). All harvesting was performed at the beginning of the stationary phase of growth.

### 4.2. Molecular Identification of Microalgae

Ribosomal DNA sequences from the *Skeletonema* strains were obtained by sequencing the small subunit (18S) and the ITS (Internal Transcribed Spacer) region including ITS1, the 5.8S unit, and ITS2. A simple DNA extraction and PCR amplification method was used to amplify the ribosomal DNA using the following primers:

18S-F (5′-ACCTGGTTGATCCTGCCAGT-3′) and 18S-R (5′-TCCTTCTGCAGGTTCACCTAC-3′)

ITS-F (5′-TTTGTACACACCGCCCGTCG-3′) and ITS-R (5′-TATGCTTAAATTCAGCGGGT-3′).

18S and ITS sequences of the genus *Skeletonema*, available in the NCBI (National Center for Biotechnology Information) database, were aligned with the target strains using Seaview software version 4.5.4 (Laboratoire de Biométrie et Biologie Evolutive, CNRS/Université de Lyon) to constitute two phylogenetic trees.

### 4.3. Pigment Extraction and Analysis

#### 4.3.1. Extraction of Pigments for Phototoxicity Screenings

Extraction was performed by adding absolute EtOH to freeze-dried paste of microalgae in the proportion of 40 mL.g^−1^. Maceration was carried out for 4 h at 20 °C under magnetic stirring. Extracts were then clarified by centrifugation (4000× *g*, 10 min, 4 °C) and filtrated with a 0.2 µm PTFE filter. Finally, extracts were dried using a rotary evaporator (35 mBar vacuum, 25 °C). All steps were performed at dark to avoid pigment photodegradation or photosensitizers activation.

#### 4.3.2. Extraction for Pigment Diversity Analysis

A specific fast extraction method was used to analyze the pigments as close as possible to the effective content of the phytoplanktonic cells. *Skeletonema* cells were harvested on a 1.2 µm glass fiber filter and immediately stored at −80 °C until extraction. Extraction was then performed by immersing the filter in 2 mL of 95% acetone followed by vortex pulsed sonication in an ultrasonic bath cooled with melting ice (10 min, 37 kHz) and storage at −20 °C overnight. Finally, the acetone extract was filtered with a 0.2 μm PTFE filter prior to immediate HPLC injection. For the analysis of the extracts identified in the phototoxicity screenings, a sampling of the ethanol extract was made just before the rotary evaporator step, and then injected into HPLC. For the analysis of fractions from the active crude extracts, samples were taken directly from the fraction collector of the semi-preparative method. All steps were performed at dark to avoid pigment photodegradation or photosensitizers activation.

#### 4.3.3. HPLC Analysis

Extracts were analyzed by HPLC-UV-DAD (1200 HPLC-UV-DAD series; Agilent Technologies, Santa Clara, CA, USA ) using an Eclipse XDB-C8 reverse phase column (4.6 mm × 150 mm, 3.5 μm particle size; Agilent Technologies) and an XDB-C8 guard column (4.6 mm × 12.5 mm, 5 µm particle size; Agilent Technologies) following the SCOR/UNESCO reference method described by Van Heukelem and Thomas (2001) [10] with slight modifications since the version described in Serive et al. (2017) [11]. Elution gradient consisted of a 30 min gradient from 95% to 5% of solvent A (70:30 methanol, 28 mM aqueous ammonium acetate), solvent B being 100% methanol. At the end of the run, an isocratic flow of 5% solvent A was maintained for 10 min rinsing. Flow rate remained at 1 mL.min^−1^ during the run. Pigment elution was acquired at 436, 450 and 405 nm. Maximum signals were used at 436 nm for chlorophyll *a* and violaxanthin, 405 nm for chlorophyll derivatives like pheophorbide *a* and pheophytin *a*, whereas 450 nm was used for all the other pigments. All spectra were acquired from 210 to 800 nm (Agilent Diode Array Detector G1315D) and ChemStation software (Agilent Technologies) was used to create a spectral library of dereplication. Pigment standards and pigment mixtures for the spectral library, calibration curves were purchased from DHI lab products (Hørsholm, Denmark). Pheophorbide *a* salt used for the evaluation of the phototoxicity of pure Pheophorbide *a* was purchased from Sigma Aldrich (St. Louis, MO, USA).

#### 4.3.4. Semi-Preparative HPLC for the Crude-Extract Fractionation

Fractionation of the *Skeletonema marinoi* crude extract was performed on a HPLC-UV-DAD system (series 1200 HPLC-UV-DAD; Agilent Technologies) using a semi-preparative column (Phenomenex Luna C18 10 mm × 250 mm, 10 µm particle size). Mobile phases were: solvent A: methanol/water (80/20); solvent B: acetonitrile/water (90/10); and solvent C: ethyl acetate. Elution gradient consisted of 100% A to 100% B in 3 min. Then, 30% B with 70% C at 35 min, 100% C at 38 min, holding isocratic until 41 min, and switching to 100% B at 43 min. Finally, A reached 100% at 45 min and remained until 47 min (rinsing). Injection volume was 500 µL and flow rate fixed at 4 mL/min and detection set at 410 and 435 nm. All peak spectra were acquired from 300 to 700 nm (Agilent Diode Array Detector G1315D). Fractions were collected according to the following retention times. F1: 0.0–2.9 min; F2: 2.9–6.0 min; F3: 6.0–10.2 min; F4: 10.2–12.2 min; F5: 12.2–20.8 min; F6: 20.8–27.5 min; F7: 27.5–32.5 min; F8: 32.5–36.0 min; F9: 36.0–37.9 min.

#### 4.3.5. LC-HRMS Analysis of Phototoxic Pigment Extracts

The fractions were analyzed by UHPLC-DAD-HRMS on a 1290 Infinity II system (Agilent Technologies) with a diode array detector (DAD, Agilent Technologies) coupled to a Q-Tof 6550 iFunnel (Agilent Technologies) equipped with a Dual Jet Stream^®^ electrospray ionization (ESI) interface operating in positive mode. Chromatographic separation was carried out on a reversed-phase C_18_ Kinetex column (100 Å, 1.7 μm, 100 × 2.1 mm, Phenomenex, LePecq, France) at 40 °C using a mobile phase composed of water (A) and 95% acetonitrile/water (B) both containing 5 mM ammonium formate and 50 mM formic acid. The flow rate was set at 0.4 mL min^−1^ and the injection volume was 5 µL. Separation was achieved using the following mobile phase gradient: start at 5% B for 1 min and rise from 5% to 95% B in 10 min, hold at 95% B for 3 min, return to the initial condition (5% B) in 0.5 min and a re-equilibration period (5% B) for 4.5 min. The DAD was set to acquire spectra in a range of wavelengths between 300 and 650 nm every 1.2 nm. The instrument was operated in full scan and targeted MS/MS modes in positive (ESI^+^) ion acquisition. The conditions of the ESI source were set as follows: source temperature, 160 °C; drying gas flow rate, 11 mL min^−1^; sheath gas temperature, 250 °C; sheath gas flow rate, 11 mL min^−1^; nebulizer, 45 psi; capillary voltage, 4.5 kV; nozzle voltage, 500 V. Mass spectra were acquired over *m/z* 100 to 1700 range with an acquisition rate of 2 spectra s^−1^. The targeted MS/MS mode over *m*/*z* 50–1700 range was set with an MS scan rate of 10 spectra s^−1^ and an MS/MS scan rate of 3 spectra s^−1^. Three fixed collision energies (20, 40 and 60 eV) were applied to obtain an overview of the fragmentation pathways. Instrument control, data processing and analysis were controlled by MassHunter software (version B08, Agilent Technologies, Santa Clara, CA, USA).

### 4.4. Evaluation of the Cytotoxicity at Obscurity and Phototoxicity after Illumination against Planktonic and Biofilm Bacteria (MIC/MBC Assays)

#### 4.4.1. Bacterial Strains and Growth

In vitro biological assays were performed on three relevant bacterial species related to acute and chronic skin diseases, including acne pathogenesis and chronic infection of wounds. These included *Staphylococcus aureus* CIP76.25, *Staphylococcus epidermidis* CIP109.562 and *Cutibacterium acnes* CIP53.117T. Bacterial culture was routinely performed using standard nutritive growth solid agar incubated at 37 °C for 24 h in the dark. *S. aureus* and *S. epidermidis* were grown in aerobiosis in liquid Tryptic Soy Broth (TSB, pancreatic casein extract 17 g·L^−1^, soy flour papaic digest 3 g·L^−1^, dextrose 2.5 g·L^−1^, NaCl 5 g·L^−1^, and K_2_HPO_4_ 2.5 g·L^−1^) and incubated overnight at 37 °C under aerobic conditions, before use for antibacterial assays. *C. acnes* was grown in anaerobic jars in liquid medium (Tryptone 30 g·L^−1^, Yeast extract 20 g·L^−1^, Glucose 5 g·L^−1^, Cysteine hydrochloride 0.5 g·L^−1^) and 25 mL hemin solution per L (0.1 g hemin chloride, 4 mL triethanolamine and 96 mL distilled water).

#### 4.4.2. Determination of MIC and MBC in Dark Condition (Cytotoxicity) and after Light Illumination (Phototoxicity) in Planktonic Bacteria

MIC and MBC were determined anaerobically for *C. acnes* or aerobically for *S. aureus* and *S. epidermidis* by a broth microdilution method in microplates with serial twofold dilutions. In a 96 well-microplate, 10^5^ CFU were incubated with different concentrations of the raw extracts. Bacteria were submitted to light exposure for 5 h at 25 J·cm^−2^ (white light), for 8 min at 37.5 J·cm^−2^ (red light) or not (dark condition). Bacteria were then incubated at 37 °C for 24 h in the dark on standard nutritive growth solid agar. MIC and MBC were determined from average values obtained from triplicate independent measurements.

#### 4.4.3. Biofilm Inhibition Assay in Dark Condition and after Light Illumination

*S. aureus* bacteria were grown for 20 h at 37 °C in TSB supplemented with glucose 25 mM. A quantity of 50 µL of different concentrations of *Skm* ethanolic extracts (from 63 to 500 µg·mL^−1^) was added to the 96-well microplate. Then, 50 µL of diluted bacterial suspension at a concentration of 4 × 10^6^ CFU.mL^−1^ was added to each well. After illumination with white light (25 J·cm^−2^), 100 µL of 2-fold concentrated TSB supplemented with glucose 50 mM were added in each well and the microplate was incubated at 37 °C for 24 h. Then, bacterial suspensions were discarded and biofilm was stained with a 0.1% (*w*/*v*) crystal violet solution for quantification. After the solubilization of crystal violet in acetic acid 30%, biofilm was quantified by measuring the absorbance at 595 nm.

#### 4.4.4. Biofilm Eradication Assay in Dark Condition and after Light Illumination

For biofilm eradication, *S. aureus* bacteria were grown for 20 h at 37 °C in TSB supplemented with glucose 25 mM, as previously described. 200 µL of diluted bacterial suspension at a concentration of 10^6^ CFU·mL^−1^ were added in each well in the 96-well microplate. Two microplates were prepared (one for dark condition and one for white light illumination) and incubated at 37 °C for 24 h, allowing biofilm formation. Then, the culture medium was discarded. After that, 100 µL of different concentrations of *Skm* ethanolic extracts (from 63 to 500 µg/mL) was added to the 96-well microplate. The microplates were incubated 2 h in dark condition, before white light irradiation. After illumination with white light (25 J·cm^−2^), 100 µL of 2-fold concentrated TSB supplemented with glucose 50 mM were added in each well and the microplate was incubated at 37 °C for 24 h. Bacterial suspensions were discarded and biofilm was stained with a 0.1% (*w*/*v*) crystal violet solution for quantification. After the solubilization of crystal violet in acetic acid 30%, biofilm was quantified by measuring the absorbance at 595 nm.

### 4.5. Cytotoxicity and Phototoxicity in Keratinocytes

Cytotoxicity and phototoxicity of the *Skm* ethanol extract were evaluated in primary Normal Human Epidermal Keratinocytes (NHEK) at obscurity or exposed to white light (25 J·cm^−2^). NHEK (Lonza, Colmar, France) were cultured in Keratinocyte Basal Medium (KBM) supplemented with KGM Gold SingleQuots (Lonza, Colmar, France), including hydrocortisone, transferrin, epinephrine, gentamicin/amphotericin GA-1000, Bovine Pituitary Extract, hEGF, and insulin. NHEK were cultured at 37 °C in 95% humidified atmosphere in the presence of 5% CO_2_. Cell viability was assessed using the 3-(4,5-dimethylthiazol-2-yl)-2,5-diphenyl tetrazolium bromide (MTT) method. 24 h prior to experiments, 100 microliters per well of a cell suspension at 0.5 × 10^5^ cells per milliliter were deposited and cultured in 96-well microplates in complete KBM culture medium. Culture medium was removed, and cells were incubated in the dark with different concentrations of *Skm* ethanol extracts (1, 5 and 10 µg·mL^−1^). After 2 h of incubation at 37 °C in the dark, cells were washed three times with PBS, and then 100 μL of fresh KBM culture medium was added in each well. One plate was exposed to a white light illumination for 5 h (25 J·cm^−2^) and then was placed at 37 °C in the dark. Another plate was kept in the dark at 37 °C without illumination (dark condition). After 24 h at 37 °C, the MTT solution was added at a final concentration of 0.5 mg·mL^−1^ in each well, and cells were incubated at 37 °C for 2 h. Then, the culture medium was harvested and replaced by 100 μL of DMSO in each well to dissolve formazan crystals. The absorbance was measured at 595 nm using a Bio-Rad iMark microplate reader.

### 4.6. Cytotoxicity and Lipogenesis Inhibition in Sebocytes

Cytotoxicity and lipogenesis inhibition by the *Skm* ethanol extract was evaluated in the SEBO662AR sebocytes cell line. Cell viability in response to Skm ethanol extract was determined using a WST-8 assay. For lipogenesis inhibition, the cells were either unstimulated or stimulated with a lipogenic mix containing dihydrotestosterone, vitamin D_3_, vitamin C, insulin, and Ca^2+^ ions (confidential concentrations, QIMA Life Sciences, Gençay, France). The stimulated cells were treated with control medium, dutasteride or cerulenin at 10 µM (lipogenesis inhibitors) or the *Skm* ethanol extract at concentrations of 1, 5 or 10 µg·mL^−1^. Cerulenin is a natural antifungal compound that hinders β-keto-acyl-ACP synthase and HMG-CoA synthetase, and dutasteride is a synthetic 4-azasteroid compound that selectively inhibits both the type I and type II isoforms of steroid 5α-reductase, both impacting steroid and fatty acid biosynthesis. Lipogenesis inhibition was evidenced by BODIPY 493/503 green labelling of lipid droplets. Cell nuclei were detected using Hoechst 33258.

### 4.7. Interspecies Comparison and Correlation between Phototoxicity and Pheophorbide a and Derivatives Content

To confirm whether the phototoxicity of the extract was related to a high pheophorbide *a* and derivatives content, three additional strains of the *Skeletonema* genus were selected for pigment extraction, dosage, phototoxicity assays and pheophorbide *a* and derivatives dosage. These included *S. subsalsum*, *S. menzelii* and *S. grethae.* A principal component analysis (PCA) of the strains according to their pigment composition allowed us to evaluate the contribution of the pigments to the phototoxicity on planktonic bacteria. Presence of pheophorbide *a* derivatives in the pigment extracts, including pheophorbide *a* isomer and 13-OH pheophorbide *a*, was determined through UPLC-HRMS determination of parent and fragment ions (see Section 4.3.5).

### 4.8. Statistical Analysis

Each experiment was conducted independently in triplicate. Quantitative data are presented as the mean ± standard error of the mean (SEM). For lipogenesis inhibition experiments, statistical analysis was performed using an unpaired Student’s *t*-test. Values of p being at least < 0.05 were considered statistically significant. Principal Component Analysis (PCA) was computed using R software with FactoMineR package (R Core Team (2023)_R: A Language and Environment for Statistical Computing. R Foundation for Statistical Computing, Vienna, Austria.

## Figures and Tables

**Table 1:** Minimal inhibitory concentration (MIC, µg·mL^−1^) of the *Skeletonema* species ethanol extracts measured after photo-illumination of bacterial planktonic cells with white light (total fluence of 25 J·cm^−2^), red light (total fluence of 37.5 J·cm^−2^) or in absence of illumination (control at dark). All experiments were performed in triplicate.

	*S. marinoi*	*S. grethae*	*S. menzelii*	*S. subsalsum*
*C. acnes*				
Dark (control)	>1000	>1000	>1000	>1000
White light	6	6	31	16
Red light	125	500	>1000	1000
*S. aureus*				
Dark (control)	250	250	>1000	>1000
White light	63	31	250	125
Red light	125	125	>1000	>1000
*S. epidermidis*				
Dark (control)	250	250	250	250
White light	16	63	63	16
Red light	63	250	250	125

**Table 2:** Minimal bactericidal concentration (MBC, µg·mL^−1^) of the *Skeletonema marinoi* ethanol extracts measured after photo-illumination of bacterial planktonic cells with white light (total fluence of 25 J·cm^−2^), or in the absence of illumination (control at dark). All experiments were performed in triplicate.

	MBC White Light (µg·mL^−1^)	MBC Dark (µg·mL^−1^)
*C. acnes*	6	>1000
*S. aureus*	63	>1000
*S. epidermidis*	63	>1000

**Table 3:** Pigment identification in the *Skeletonema marinoi* ethanol extract was conducted considering polarity and high-resolution mass spectrometric data. Mass differences (ppm) were compared between theoretical exact and measured masses. It should be noted that retention times (Rt) varied between RP-HPLC and UPLC-HRMS analyses. “Max. Abs” refers to maximal absorption wavelengths. Absorption spectra, maximal absorption wavelengths, band III/II ratios for carotenoids, Sorets for porphyrins were also considered to confirm the pigment identification.

Molecule	Formula	Ion	Theoretical Monoisotopic Mass (*m*/*z*)	Peak Area (×10^6^)	RT (min)	Score (%)	Experimental Monoisotopic Mass (*m*/*z*)	Error (ppm)
Pheophorbide *a*	C_35_H_37_O_5_N_4_	[M + H]^+^	593.2758	303	11.18	98.5	593.2775	2.79
		[M + K]^+^	631.2312				631.2343	4.91
		[M + Na]^+^	615.2578				615.2579	0.16
Pheophorbide *a* isomer	C_35_H_37_O_5_N_4_	[M + H]^+^	593.2758	127	11.43	97.3	593.2760	0.26
		[M + K]^+^	631.2312				631.2292	−3.17
		[M + Na]^+^	615.2578				ND	
all-*trans*-Fucoxanthin	C_42_H_58_O_6_	[M + H]^+^	659.4306	184	10.88	97.6	659.4307	0.13
		[M + H − H_2_O]^+^	641.4201				641.4201	0.08
		[M + Na]^+^	681.4126				681.4134	1.23
*cis*-Fucoxanthin	C_42_H_58_O_6_	[M + H]^+^	659.4306	359	11.52	95.8	659.4324	2.71
		[M + H − H_2_O]^+^	641.4201				641.4184	−2.57
		[M + Na]^+^	681.4126				681.4147	3.14
13-OH pheophorbide *a*	C_35_H_36_N_4_O_6_	[M + H]^+^	609.2708	465	11.01	99.4	609.2709	0.16
		[M + K]^+^	647.2266				647.2386	18.54
		[M + Na]+	631.2527				631.2530	0.48

**Table 4:** Bacterial growth inhibition induced by *Skm* ethanol extract subfractions under varying light conditions: white light illumination, red light illumination, and at dark. Subfractions F3j and F4b exhibited bactericidal effects under both light illumination and at dark, indicating the presence in these subfractions of bactericidal molecules independently of photoactivation. In contrast, subfractions F5f and F5g displayed bactericidal activity exclusively under white or red light illumination, highlighting their phototoxic activity. The notation (+) indicate that the subfraction is bactericidal in the tested conditions.

Sub-Fraction	*S.aureus* Growth Inhibition under White Light	*S.aureus* Growth Inhibition under Red Light	*S.aureus* Growth Inhibition at Dark (Control)
F3a to F3i	-	-	-
F3j	+	+	+
F3k to F3n	-	-	-
F4a	-	-	-
F4b	+	+	+
F4c	-	-	-
F5a to F5e	-	-	-
F5f	+	+	-
F5g	+	+	-
F5h to F5n	-	-	-

**Table 5:** Quantification of pigment content in the total ethanolic extracts of *Skeletonema marinoi*, *Skeletonema grethae*, *Skeletonema subsalsum*, and *Skeletonema menzelii*, expressed as milligrams per gram of freeze-dried biomass. Pigment analysis was conducted using the HPLC-UV/DAD Van Heukelem reference dereplication method. The *Skm* strain employed in this study demonstrated a notable ability to biosynthesize pheophorbide *a*.

	*S. marinoi* IFR-855	*S. grethae* CCAP 1077/3	*S. subsalsum * SAG 8.94	*S. menzelii* CCMP 787
Chlorophyll c2	0.36	0.20	0.05	0.56
Chlorophyll c1	0.17	0.12	0.03	Undetected
Chlorophyll *a*	2.49	3.22	6.36	5.05
Pheophorbide *a*	5.94	0.50	Undetected	Undetected
*cis*-Fucoxanthin	0.06	Undetected	0.05	0.12
All Trans-Fucoxanthin	1.98	1.52	1.73	4.64
Diadinoxanthin	0.07	0.36	0.73	0.63
Diatoxanthin	1.18	0.18	0.49	0.49
Violaxanthin	Undetected	Undetected	0.02	Undetected
Zeaxanthin	0.10	0.04	0.04	Undetected
β-Carotene	0.33	0.16	0.20	0.15

**Table 6:** Quantification of mean pigment content in the biomass of four strains of *Skeletonema marinoi* grown in triplicate under homogenous controlled conditions in the phenotyping bench, expressed as milligrams per gram of dried biomass. A fast extraction method was used to analyze the pigments as close as possible to the effective content of the living cell (see Section 4.3.2). Pigment analysis was conducted using the HPLC-UV/DAD Van Heukelem reference dereplication method.

	*S. marinoi* IFR-855	*S. marinoi* AC-714	*S. marinoi* CCAP 1077/5	*S. marinoi* SAG 19.99
Chlorophyll *c*2	0.143 ± 0.003	0.207 ± 0.003	0.336 ± 0.077	0.510 ± 0.115
Chlorophyll *c*1	0.118 ± 0.002	0.200 ± 0.004	0.386 ± 0.049	0.394 ± 0.066
Chlorophyll *a*	0.135 ± 0.018	0.493 ± 0.126	0.302 ± 0.065	0.416 ± 0.042
Pheophorbide *a*	1.968 ± 0.081	1.971 ± 0.180	1.483 ± 0.101	1.222 ± 0.213
Fucoxanthin	0.085 ± 0.021	1.615 ± 0.068	0.215 ± 0.102	0.532 ± 0.147
Diadinoxanthin	0.376 ± 0.009	0.237 ± 0.005	0.086 ± 0.030	0.367 ± 0.084
Diatoxanthin	0.064 ± 0.002	0.167 ± 0.008	0.055 ± 0.017	0.079 ± 0.020
Zeaxanthin	0.023 ± 0.006	0.006 ± 0.001	0.025 ± 0.002	0.000 ± 0.000
β,β-Carotene	0.021 ± 0.002	0.025 ± 0.001	0.031 ± 0.008	0.071 ± 0.017
Ratio Pheo *a*/Chl *a*	14.578	3.998	4.911	2.938

## Data Availability

The data presented in this study are available on request from the corresponding author (Jean-Baptiste Bérard, jean.baptiste.berard@ifremer.fr).

## Supplementary Materials

The following supporting information can be downloaded at: https://www.mdpi.com/article/10.3390/md22080343/s1, Supplementary Data S1: 18S and ITS molecular analysis of IFR-855, AC-714, CCAP 1077/5, and SAG 19.99 *Skeletonema marinoi* strains.
