# Supplementary material for: Phototoxicity of the Ethanolic Extract of Skeletonema marinoi for the Dermocosmetic Improvement of Acne
Source: Mar Drugs. 2024 Jul 27;22(8):343. doi: 10.3390/md22080343 (PMC11355829; doi:10.3390/md22080343)
Supplement: Supplementary file 1 [file marinedrugs-22-00343-s001.zip › marinedrugs-3126852-supplementary.pdf]

Supplementary data S1 : 18S and ITS molecular analysis of IFR-855, AC-714, CCAP 1077/5, and SAG 19.99 *Skeletonema marinoi* strains.

Ribosomal DNA sequences from *Skeletonema* strains IFR-855, AC-714, CCAP 1077/5 and SAG 19.99 were obtained by sequencing the small subunit (18S) and the ITS (Internal Transcribed Spacer) region comprising ITS1, 5.8S and ITS2.

A simple DNA extraction and PCR amplification method was used to amplify ribosomal DNA using the following primers:

18S-F (5'-ACCTGGTTGATCCTGCCAGT-3') and 18S-R (5'-TCCTTCTGCAGGTTCACCTAC-3')

ITS-F (5'-TTTGTACACACCGCCCGTCG-3') and ITS-R (5'-TATGCTTAAATTCAGCGGGT-3')

18S and ITS sequences from the *Skeletonema* genus, available in the NCBI (National Center for Biotechnology Information) database, were aligned with the target strains using Seaview version 4.5.4 software (Laboratoire de Biométrie et Biologie Evolutive, CNRS/Université de Lyon) to form the phylogenetic trees.

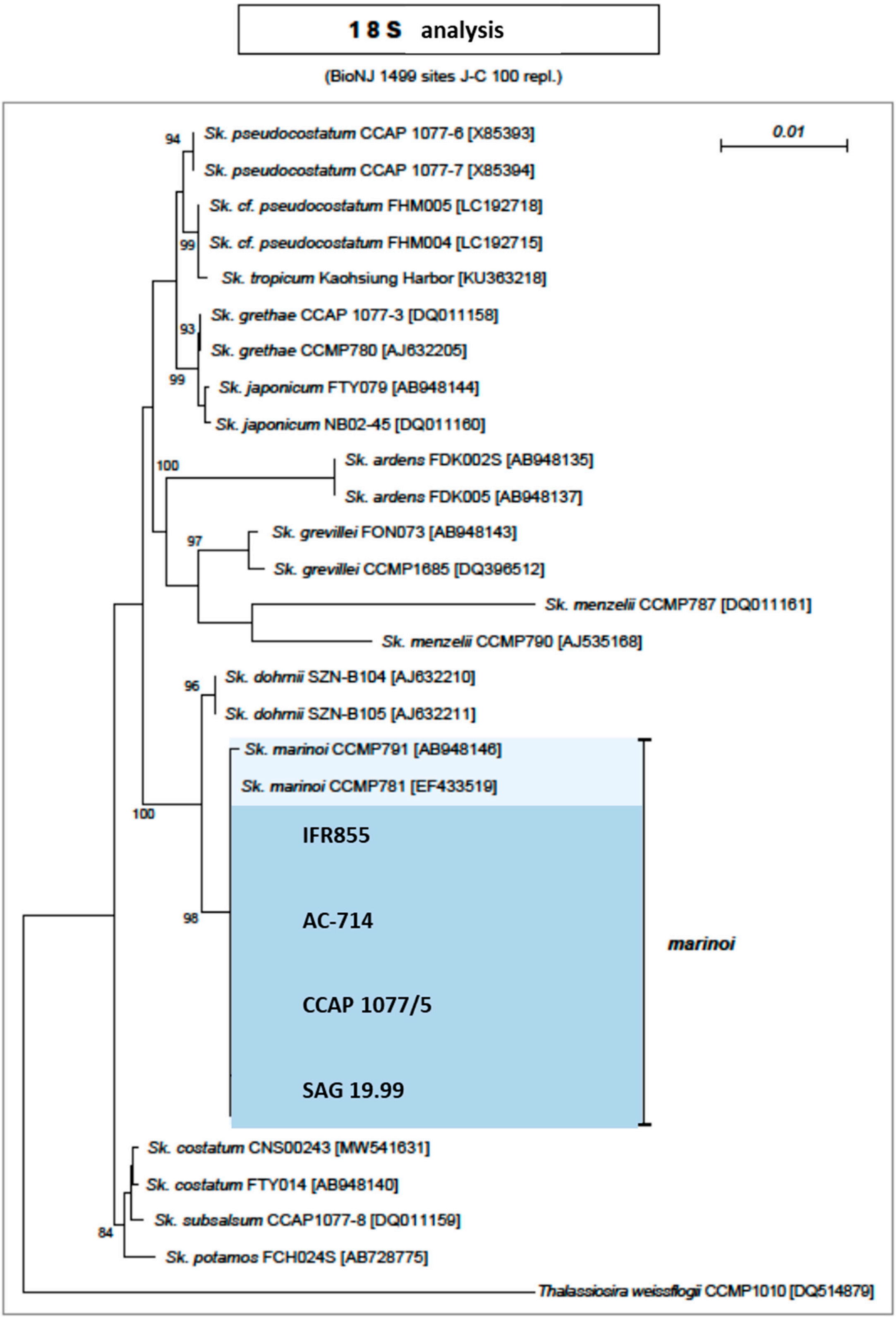

Seaview 18S SK PHASMA+NCBI (107-1603bp)-BioNJ_tree Tue Nov 09 10:25:09 2021

ITS analysis

(BioNJ 554 sites J-C 100 repl.)

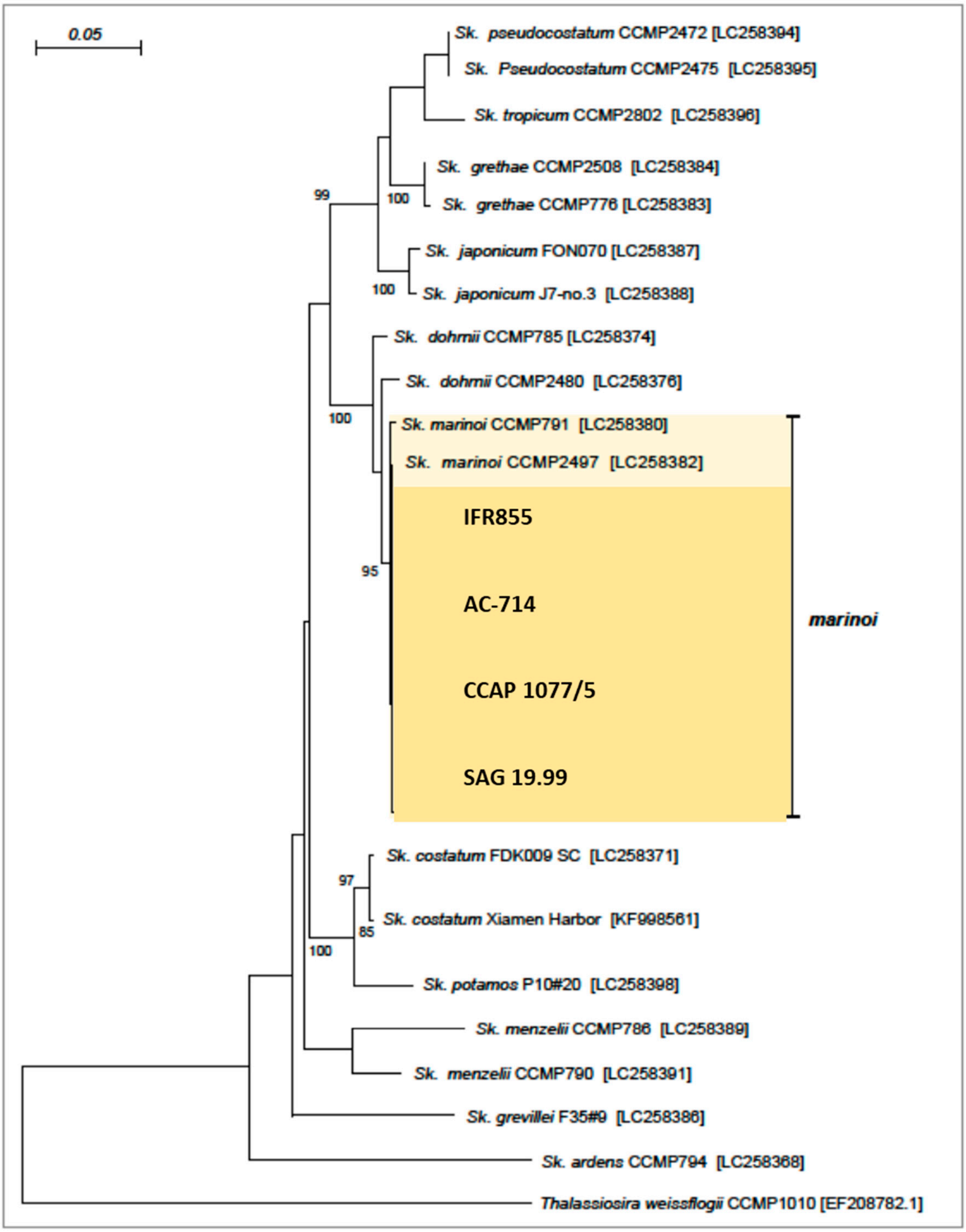

Seaview ITS PHASMA+NCBI (113-829)-BioNJ_tree Tue Nov 09 16:02:20 2021

Identity Matrix for 18S sequences

	(%)	**855**	**AC714**	**CCAP1077-5**	**SAG19.99**	*Sk. marinoi* CCMP791 [AB948146]	*Sk. marinoi* CCMP781 [EF433519]
Sk. marinoi	**855**	**ID**	**100**	**100**	**100**	99.9	100
	**AC714**	**100**	**ID**	**100**	**100**	99.9	100
	**CCAP1077-5**	**100**	**100**	**ID**	**100**	99.9	100
	**SAG19.99**	**100**	**100**	**100**	**ID**	99.9	100
	Sk. marinoi CCMP791 [AB948146]	99.9	99.9	99.9	99.9	ID	99.9
	Sk. marinoi CCMP781 [EF433519]	100	100	100	100	99.9	ID
	Sk. dohrnii SZN-B104 [AJ632210]	99.6	99.6	99.6	99.6	99.6	99.6
	Sk. dohrnii SZN-B105 [AJ632211]	99.6	99.6	99.6	99.6	99.6	99.6
	Sk. pseudocostatum CCAP 1077-6 [X85393]	98.9	98.9	98.9	98.9	98.8	98.9
	Sk. pseudocostatum CCAP 1077-7 [X85394]	98.9	98.9	98.9	98.9	98.8	98.9
	Sk. cf. pseudocostatum FHM004 [LC192715]	98.8	98.8	98.8	98.8	98.8	98.8
	Sk. cf. pseudocostatum FHM005 [LC192718]	98.8	98.8	98.8	98.8	98.7	98.8
	Sk. costatum CNS00243 [MW541631]	98.8	98.8	98.8	98.8	98.8	98.8
	Sk. costatum FTY014 [AB948140]	98.8	98.8	98.8	98.8	98.8	98.8
	Sk. grethae CCAP 1077-3 [DQ011158]	98.8	98.8	98.8	98.8	98.7	98.8
	Sk. japonicum FTY079 [AB948144]	98.8	98.8	98.8	98.8	98.7	98.8
	Sk. tropicum Kaohsiung Harbor [KU363218]	98.8	98.8	98.8	98.8	98.7	98.8
	Sk. grethae CCMP780 [AJ632205]	98.7	98.7	98.7	98.7	98.6	98.7
	Sk. japonicum NB02-45 [DQ011160]	98.7	98.7	98.7	98.7	98.6	98.7
	Sk. potamos FCH024S [AB728775]	98.7	98.7	98.7	98.7	98.7	98.7
	Sk. subsalsum CCAP1077-8 [DQ011159]	98.7	98.7	98.7	98.7	98.6	98.7
	Sk. grevillei FON073 [AB948143]	98.4	98.4	98.4	98.4	98.4	98.4
	Sk. grevillei CCMP1685 [DQ396512]	98.3	98.3	98.3	98.3	98.2	98.3
	Sk. ardens FDK002S [AB948135]	97.8	97.8	97.8	97.8	97.7	97.8
	Sk. ardens FDK005 [AB948137]	97.8	97.8	97.8	97.8	97.7	97.8
	Sk. menzelii CCMP790 [AJ535168]	97.3	97.3	97.3	97.3	97.2	97.3
	Sk. menzelii CCMP787 [DQ011161]	96.0	96.0	96.0	96.0	96.0	96.0

Identity Matrix for ITS sequences

	(%)	**855**	**AC714**	**SAG19.99**	**CCAP1077-5**	*Sk. marinoi* ccmp2497 [LC258382]	*Sk. marinoi* ccmp791 [LC258380]
Sk. marinoi	**855**	**ID**	**100**	**100**	**99.8**	100	99.6
	**AC714**	**100**	**ID**	**100**	**99.8**	100	99.6
	**SAG19.99**	**100**	**100**	**ID**	**99.8**	100	99.6
	**CCAP1077-5**	**99.8**	**99.8**	**99.8**	**ID**	99.8	99.4
	Sk. marinoi ccmp2497 [LC258382]	100	100	100	99.8	ID	99.6
	Sk. marinoi ccmp791 [LC258380]	99.6	99.6	99.6	99.4	99.6	ID
	Sk. dohrnii ccmp785 [LC258374]	97.8	97.8	97.8	97.6	97.8	97.4
	Sk. dohrnii ccmp2480 [LC258376]	97.6	97.6	97.6	97.5	97.6	97.6
	Sk. costatum Xiamen Harbor [KF998561]	91.0	91.0	91.0	90.8	91.0	91.0
	Sk. japonicum J7-no.3 [LC258388]	90.9	90.9	90.9	90.7	90.9	90.9
	Sk. japonicum FON070 [LC258387]	90.7	90.7	90.7	90.5	90.7	90.7
	Sk. costatum NMBguh004-2 [MZ823505]	90.6	90.6	90.6	90.5	90.6	90.6
	Sk. grethae ccmp2508 [LC258384]	90.5	90.5	90.5	90.3	90.5	90.5
	Sk. grethae ccmp776 [LC258383]	90.2	90.2	90.2	90	90.2	90.2
	Sk. costatum FDK009 SC [LC258371]	89.7	89.7	89.7	89.5	89.7	89.7
	Sk. pseudocostatum ccmp2472 [LC258394]	89.5	89.5	89.5	89.3	89.5	89.5
	Sk. pseudocostatum ccmp2475 [LC258395]	89.5	89.5	89.5	89.3	89.5	89.5
	Sk. potamos P10#20 [LC258398]	88.7	88.7	88.7	88.5	88.7	88.7
	Sk. tropicum ccmp2802 [LC258396]	88.4	88.4	88.4	88.2	88.4	88.4
	Sk. menzelii ccmp790 [LC258391]	87.6	87.6	87.6	87.5	87.6	87.6
	Sk. menzelii ccmp786 [LC258389]	85.6	85.6	85.6	85.8	85.6	85.6
	Sk. grevillei F35#9 [LC258386]	85.2	85.2	85.2	85.1	85.2	85.6
	Sk. ardens ccmp794 [LC258368]	77.0	77.0	77.0	77.0	77.0	77.0
